# Molecular evidence for the occurrence of a new sibling species within the *Anopheles (Kerteszia) cruzii *complex in south-east Brazil

**DOI:** 10.1186/1475-2875-9-33

**Published:** 2010-01-26

**Authors:** Luísa DP Rona, Carlos J Carvalho-Pinto, Alexandre A Peixoto

**Affiliations:** 1 Laboratório de Biologia Molecular de Insetos, Instituto Oswaldo Cruz, FIOCRUZ, Av. Brasil 4365, Rio de Janeiro 21045-900, RJ, Brazil; 2 Departamento de Microbiologia e Parasitologia, CCB, Universidade Federal de Santa Catarina, Florianópolis 88040-970, SC, Brazil

## Abstract

**Background:**

*Anopheles cruzii *(Diptera: Culicidae) has long been known as a vector of human and simian malaria parasites in southern and south-eastern Brazil. Previous studies have provided evidence that *An. cruzii *is a species complex, but the status of the different populations and the number of sibling species remains unclear. A recent analysis of the genetic differentiation of the *timeless *gene among *An. cruzii *populations from south and south-east Brazil has suggested that the population from Itatiaia, Rio de Janeiro State (south-east Brazil), is in a process of incipient speciation.

**Methods:**

A ~180 bp fragment of *cpr*, a gene encoding the NADPH-cytochrome P450 reductase, an enzyme involved in metabolic insecticide resistance and odorant clearance in insects, was used in this study as a molecular marker to analyse the divergence between five *An. cruzii *populations from south and south-east Brazil.

**Results:**

Analysis of the genetic differentiation in the *cpr *gene revealed very high *F_ST _*values and fixed differences between Itatiaia and the other four populations studied (Florianópolis, Cananéia, Juquitiba and Santa Teresa). In addition, the data also provided preliminary evidence that seems to indicate the occurrence of two sympatric sibling species in Itatiaia.

**Conclusions:**

Population genetics analysis of *An. cruzii *samples from different localities using a fragment of the *cpr *gene suggests that the Itatiaia sample represents at least one new sibling species in this complex.

## Background

*Anopheles cruzii *has long been known as a vector of human and simian malaria parasites in southern and south-eastern Brazil [1,2]. This species, which belongs to the subgenus *Kerteszia*, is found from the coast of Rio Grande do Sul State in southern Brazil to Sergipe State in north-eastern Brazil [3,4], all along the Brazilian Atlantic forest. This forest provides an excellent environment for *An. cruzii*, since it is an ecosystem abundant in bromeliads, the larval habitat for this anopheline [2,5,6].

The possibility that *An. cruzii *could represent more than a single species was first supported by morphological differences observed among populations from the states of Santa Catarina and Rio de Janeiro [3]. Later it was revealed that *An. cruzii *is polymorphic for chromosome rearrangements [7,8]. Differences in inversion frequencies and *X *chromosome banding patterns from populations in south-eastern and southern Brazil have suggested a process of incipient speciation [9,10]. Malafronte *et al *[11] compared sequences of ITS2 (Internal Spacer Region 2) from several *An. cruzii *populations from south and south-east Brazil and found differences between sequences from different localities, although they considered premature to conclude based on their results that there are distinct sibling species in the areas investigated. Similar results were observed by Calado *et al *[12], using PCR-RAPD and PCR-RFLP of the ITS2 region.

Finally, isoenzyme analysis indicated two genetically isolated groups, one from Bahia State (north-eastern Brazil), and the other from south-eastern and southern Brazil (Rio de Janeiro, São Paulo and Santa Catarina States) [13]. Supporting the isoenzyme results, analysis of the molecular polymorphism and genetic differentiation of the *timeless *gene among Brazilian populations of *An. cruzii *also indicated two cryptic species, one occurring in the north-east (Bahia State) and another in south and south-east Brazil (Espírito Santo, Rio de Janeiro, São Paulo and Santa Catarina States). In addition, the *timeless *gene sequences also suggested that populations from the south and south-east regions might also constitute different incipient species within this complex in Brazil [14]. Since previous *X *chromosome analyses suggested the existence of sibling species in these Brazilian regions [9,10], it would be interesting to analyse the same populations with an *X*-linked molecular marker to see whether a higher level of differentiation is found.

The gene encoding the NADPH-cytochrome P450 reductase (CPR) has been cloned from several insect species [15-17]. In *Anopheles gambiae*, this protein is encoded by a single copy gene located on the *X *chromosome [17,18]. Previous studies have shown that the cytochrome P450 gene family, which is involved in metabolic insecticide resistance, requires CPR to function [19,20]. Additionally, knockdown of CPR expression increases *An. gambiae *sensitivity to the insecticide permethrin [21]. Another putative function associated with CPR in insects is odorant clearance, since the *cpr *gene is highly expressed in the antennae of the fruit fly *Drosophila melanogaster *and more specifically at the base of olfactory sensilla in the moth *Mamestra brassicae *[16,22]. Olfactory cues are important environmental stimuli affecting mosquito behaviour, playing significant roles in the location of food sources, mates and oviposition sites [23]. Therefore, genes involved in the regulation of antennal response to pheromones can be potentially important in maintaining sexual isolation between closely related species, and in this case, the CPR would be an interesting molecular marker for population studies of the *An. cruzii *complex.

In this study, an analysis of intraspecific variability and genetic divergence among five Brazilian populations of *An. cruzii *was carried out using a fragment of the *cpr *gene.

## Methods

**Figure 1:**
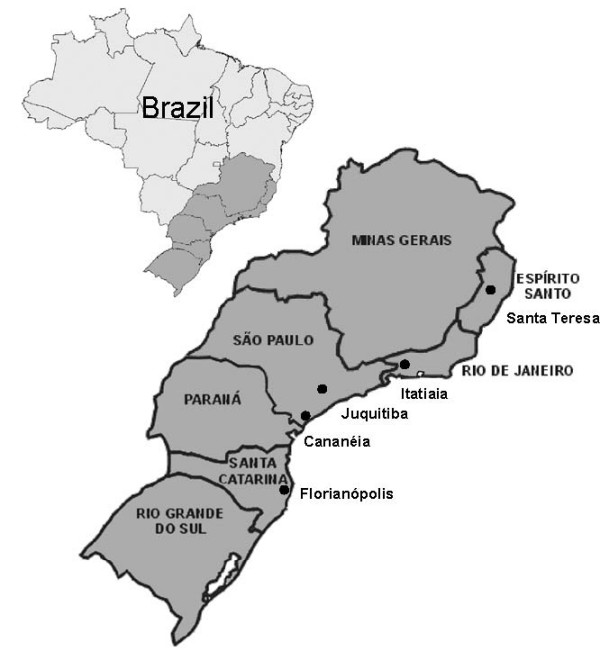
***Anopheles cruzii *populations**. Localities where the five Brazilian *An. cruzii *populations were collected (Source: IBGE).

The mosquitoes used in this study were females captured at different localities along the Brazilian Atlantic forest and identified on the basis of their morphology according to Consoli and Lourenço-de-Oliveira [4]. A total of 56 individuals were used for the molecular analysis: 14 from Florianópolis, Santa Catarina State (SC) (27°31'S/48°30'W), 12 from Cananéia, São Paulo State (SP) (25°01'S/47°55'W), 12 from Juquitiba, São Paulo State (SP) (23°57'S/47°03'W), 11 from Itatiaia, Rio de Janeiro State (RJ) (22°27'S/44°36'W) and 7 from Santa Teresa, Espírito Santo State (ES) (19°56'S/40°35'W) (Figure 1).

For the isolation of a fragment of the *An. cruzii cpr *gene, initially a pair of degenerated primers was designed based on conserved regions of the CPR proteins from *Drosophila melanogaster*,* Drosophila pseudoobscura*, *Musca domestica*,* Aedes aegypti *and *An. gambiae *(Table 1 and Additional file 1). These primers, named here 5'Cpr01deg and 3'Cpr01deg, were used in PCR with *An. cruzii *genomic DNA extracted according to Jowett [24]. PCR was carried out with an Eppendorf Mastercycler^® ^thermocycler using the following conditions: 15 cycles at 94°C for 60 s, 50°C (decreasing 1°C/cycle) for 90 s and 72°C for 60 s, followed by 20 cycles of 94°C for 60 s, 50°C for 90 s and 72°C for 60 s. PCR products were then purified and cloned using Zero Blunt TOPO PCR cloning kit (Invitrogen).

**Table 1:** Sequence of primers used to amplify the *cpr *gene fragments

Primer names	Sequence of primers (5' → 3')
5'Cpr01deg	ATGAARGGNATGGTNGCNGA (forward)
3'Cpr01deg	ATCCARTCRTARAAYTCCAT (reverse)
5'cpr01ancruzii	AGTGTAATATGGTAAGCG (forward)
3'cpr01ancruzii	GATTTCTCGATGTCTTTCAG (reverse)

Degenerate and specific primers used to amplify the *cpr *gene fragments in all *Anopheles cruzii *populations.

Sequencing of positive clones was carried out in an ABI Prism 3730 DNA sequencer (PDTIS-FIOCRUZ DNA sequencing facility) using the ABI Prism Big Dye Terminator Cycle Sequencing Ready Reaction kit (Applied Biosystems). The identity of the cloned fragments was confirmed by BlastX analysis using the GenBank [25]. Based on these initial sequences, two new specific primers named 5'cpr01ancruzii and 3'cpr01ancruzii (Table 1 and Additional file 1) were designed to amplify a ~180 bp fragment of the *An. cruzii cpr *gene from individual mosquitoes of the different localities listed above. This short fragment includes an intron of variable size (see below) and two small segments of the flanking exons (Additional file 1). PCR amplification using the specific primers was carried out for 35 cycles at 94°C for 30 s, 50°C for 60 s and 72°C for 90 s using the proofreading *Pfu *DNA polymerase (Biotools). PCR fragments were cloned using either Zero Blunt TOPO PCR cloning kit (Invitrogen) or pMOS *Blue *vector blunt-ended cloning kit (GE Healthcare) and at least eight clones of each mosquito were sequenced.

Sequences were edited and in most cases consensus sequences representing the two alleles were generated. In a number of individuals only one haplotype was observed among the eight sequences and in these cases the mosquitoes were classified as homozygotes. The probability of incorrectly classifying a heterozygote as a homozygote individual with this procedure is less than 1%. Eight homozygotes were found in Florianópolis, four in Cananéia, seven in Juquitiba, three in Itatiaia and two in Santa Teresa. The sequences from homozygote mosquitoes were duplicated prior to analysis. However, the analysis was also carried out without duplicating the homozygote sequences with similar results.

The *cpr *sequences were aligned using ClustalX software [26] and the population genetics analysis was carried out using DNASP4.0 [27], P_RO_S_EQ_v 2.91 [28] and Arlequin 3.0 [29] softwares. The haplotype network was estimated using TCS1.21 [30].

## Results

### Polymorphism and divergence among *An. cruzii *populations

**Figure 2:**
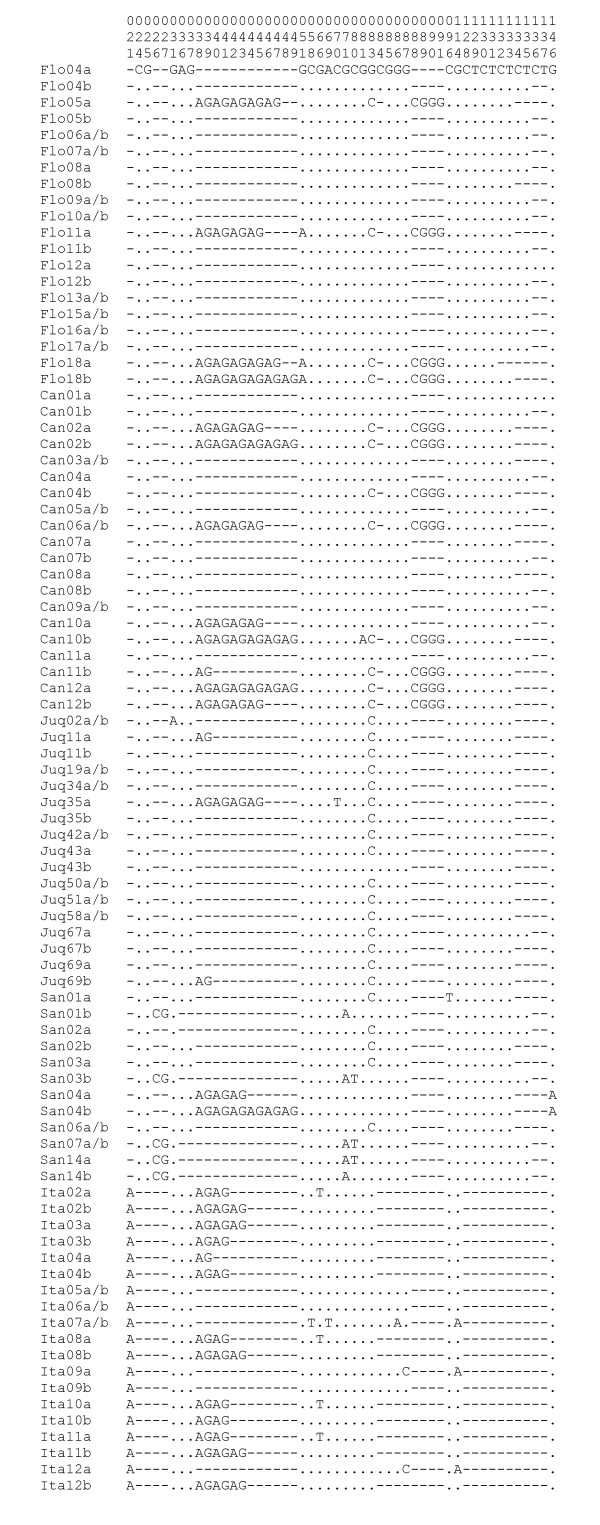
**Alignment of the variable sites in the *cpr *fragment of *An. cruzii***. Alignment of the variable positions in the DNA sequences from the *cpr *gene fragment from all populations of *An. cruzii *analysed. The sequences of homozygote individuals were grouped and are represented as a/b. Dots represent the identity of the first nucleotide sequence and asterisks represent the identity of all sequences. Flo: individuals from Florianópolis; Can: Cananéia; Juq: Juquitiba; Ita: Itatiaia; San: Santa Teresa.

A total of 112 sequences were obtained (28 from Florianópolis, 24 from Cananéia, 24 from Juquitiba, 22 from Itatiaia and 14 from Santa Teresa). The sequences were submitted to the GenBank (accession numbers: GU072619 - GU072730). An alignment of the variable sites in shown in Figure 2 (an alignment of the whole sequences is presented in Additional file 2). The small segments of the flanking exons in this *cpr *fragment are totally conserved among the five populations analysed. Therefore, all base substitutions occurred in the intron which also shows a number of indels, including three polymorphic dinucleotide repetitions (Figure 2 and Additional file 2). Table 2 shows the number of copies of each dinucleotide repeat in all *An. cruzii *populations. In Itatiaia only one repeat of CG dinucleotide was found, while in the other four populations there are two or three repeats. Similar pattern was observed for the CT repeat which shows four copies in Itatiaia while there are six to nine in the other populations (Table 2).

**Table 2:** Number of each dinucleotide repeats in *An. cruzii *populations.

			N° of alleles in each population			
		**Florianópolis**	**Cananéia**	**Juquitiba**	**Santa Teresa**	**Itatiaia**

CG	01 repeat	-	-	-	-	22
	02 repeats	28	24	24	08	-
	03 repeats	-	-	-	06	-

AG	02 repeats	-	-	-	07	-
	03 repeats	24	15	21	05	09
	04 repeats	-	01	02	-	01
	05 repeats	-	-	-	-	07
	06 repeats	-	-	-	01	05
	07 repeats	01	05	01	-	-
	08 repeats	02	-	-	-	-
	09 repeats	01	03	-	01	-

CT	04 repeats	-	-	-	-	22
	06 repeats	01	-	-	-	-
	07 repeats	04	13	23	07	-
	08 repeats	21	10	01	07	-
	09 repeats	02	01	-	-	-


Location of each type of dinucleotide repeat (see Additional file 2): CG repeats at positions 22 to 27, uninterrupted AG repeats (excluding flanking repeats with point mutations) at positions 32 to 49 and CT repeats at positions 120 to 137.

Table 3 shows the pair-wise estimates of population differentiation between the *An. cruzii *populations. Because this *cpr *fragment contains a number of indels, the *F*_*ST *_values were calculated in two different ways. In the first one (*F*_*ST *(1)_) the gaps were treated as single mutations in the analysis performed with the P_RO_S_EQ_v 2.91 software. In the second one (*F*_*ST*(2)_), the Arlequin 3.0 software was used to calculate the differentiation values considering the three types of dinucleotide repeats as microsatellite loci. In all cases the *F_ST _*values were significant and the two types of estimates showed similar results in most cases. Very high *F*_*ST *_values (ranging from ~0.6 to 0.8) were found between Itatiaia and the other populations. Albeit significant, the pair-wise *F_ST _*values in the comparisons among the samples of Florianópolis, Cananéia and Santa Teresa are usually under 0.2, while those between Juquitiba and the other three populations are moderately high.

**Table 3:** Genetic differentiation between *An. cruzii *populations

Populations	*F*_*ST*(1)_	*P*-value	*F*_*ST*(2)_	*P*-value	*D_xy_*	*D_a_*	*S_s_*	*S_f_*	*S_1_*	*S_2_*
1. Florianópolis × Cananéia	0.0845	0.013	0.1445	0.004	0.0220	0.0029	07	00	03	02
2. Cananéia × Santa Teresa	0.1811	0.000	0.1827	0.007	0.0339	0.0061	04	00	05	07
3. Florianópolis × Santa Teresa	0.1575	0.000	0.3196	0.000	0.0307	0.0075	04	00	06	07
4. Cananéia × Juquitiba	0.2673	0.000	0.2008	0.000	0.0216	0.0064	06	00	03	02
5. Santa Teresa × Juquitiba	0.2661	0.000	0.4267	0.000	0.0296	0.0104	04	00	07	04
6. Florianópolis × Juquitiba	0.3616	0.000	0.5238	0.000	0.0231	0.0126	05	00	05	03
7. Cananéia × Itatiaia	0.6105	0.000	0.6627	0.000	0.0597	0.0362	03	04	06	07
8. Santa Teresa × Itatiaia	0.6557	0.000	0.5980	0.000	0.0630	0.0355	02	03	09	08
9. Florianópolis × Itatiaia	0.6812	0.000	0.7355	0.000	0.0564	0.0375	02	04	08	08
10. Juquitiba × Itatiaia	0.7710	0.000	0.8078	0.000	0.0618	0.0470	02	04	06	08

*F*_*ST*_, pair-wise estimates of population differentiation (see text for more details); *P*-value, significance of *F*_*ST *_values (evaluated by 1,000 random permutations). *D*_*xy*_, average number of nucleotide substitutions per site between populations [39]; *D_a_*, number of net nucleotide substitutions per site between populations [39]. *S*_1_, number of polymorphic sites exclusive to the first population shown in the first column. *S*_2_, number of polymorphic sites exclusive to the second population shown in the first column. *S_s_*, number of shared polymorphisms between the two populations. *S*_f_, number of fixed differences between the two populations. These values were calculated with P_RO_S_EQ _v 2.91 [28] using the alignment shown in Additional file 2 and considering the gaps as single mutations.

Table 3 also shows the average number of nucleotide substitutions per site (*D_xy_*), the number of net nucleotide substitutions per site between populations (*D_a_*) and the distribution of the four mutually exclusive categories of segregating sites observed in each comparison: the number of exclusive polymorphisms for each population (*S_1 _*and *S_2_*), the number of shared polymorphisms (*S_s_*) and the number of fixed differences (*S_f_*). As in the case of the *F_ST_*, the highest *D_xy _*and *D_a _*values are those involving the Itatiaia population. In addition, this sample shows few shared polymorphisms and it is the only one presenting fixed differences in comparisons with the other populations.

### Genealogy of the *An. cruzii cpr *sequences

**Figure 3:**
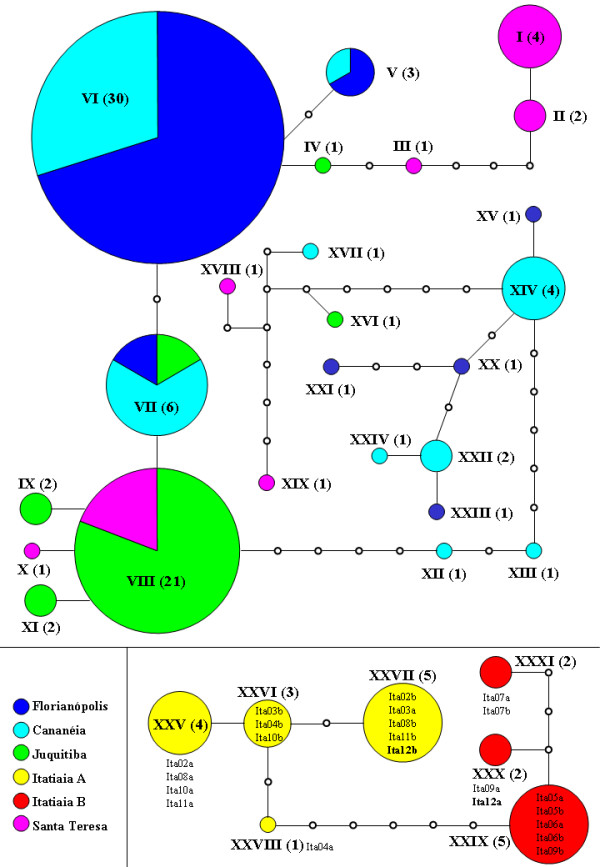
**Haplotype network of *cpr *sequences**. Each colour represents one population of *An. cruzii*. Each circle represents a different haplotype with size proportional to its relative frequency. Haplotype numbers are given in Roman and the number of sequences of each haplotype is given in brackets. The small white circles represent missing intermediates and the lines connecting the haplotypes represent one mutational step between two observed haplotypes. Each individual of Itatiaia population is discriminated next to the respective haplotype.

A network of genealogical relationships of *An. cruzii *haplotypes was estimated using the method of Templeton *et al *[31] available in the TCS programme (Figure 3). Gaps were treated as a 5^th ^state. A network was also estimated ignoring the gaps, but in this case much of the divergence among the sequences was lost and the network was not informative. The haplotype network shows that the Itatiaia population is clearly separated in an isolated group. A less clear separation was found among the sequences of the other populations.

### Divergence between Itatiaia A and Itatiaia B

Inspection of the *cpr *sequences presented in Figure 2 and Additional file 2 suggests that the Itatiaia sample might include two different sets of individuals. Based on the number of uninterrupted AG repeats between positions 32 to 49 (Figure 2) the Itatiaia population was divided in two groups: the first one, called henceforth Itatiaia A, has more than three AG repeats (04 to 06 repeats) and the second, called henceforth Itatiaia B, has exactly three AG repeats (Table 2). According to this classification the individuals Ita2, Ita3, Ita4, Ita8, Ita10 and Ita11 belong to Itatiaia A (genotype "4-6/4-6"), the mosquitoes Ita5, Ita6, Ita7 and Ita9 belong to Itatiaia B (genotype "3/3") and individual Ita12 is the only "hybrid" between the two groups (genotype "3/4-6"). Therefore, the Itatiaia sample is not in Hardy-Weinberg equilibrium (*X*^2 ^= 7.24; d.f. = 1; P < 0.01) suggesting the possibility that two sympatric sibling species might exist in this locality. The separation between the two groups is also evident in Figure 3. Besides, the *F_ST _*value (considering gaps as single mutations) between Itatiaia A and B is quite large (0.6678) and highly significant (P < 0.001) despite the small sample sizes.

Finally, to test the hypothesis that the Itatiaia population might include two different sympatric sibling species, the recently published *timeless *data [14] from the same sample were reanalysed. As for the *cpr *data, the *timeless *sequences were divided into Itatiaia A (Ita2, Ita3, Ita4, Ita8, Ita10 and Ita11) and Itatiaia B (Ita5, Ita6, Ita7 and Ita9). The *timeless *gene also suggests that the sequences might belong to two different sibling species with a *F_ST _*value (0.3418) that is highly significant (P < 0.001) and the occurrence of two fixed differences. There is also a clear separation between the Itatiaia A and B *timeless *sequences in a haplotype network (Additional file 3).

## Discussion

The *X *chromosome seems to be enriched in genes that cause reproductive isolation between species in the genus *Drosophila *[32]. In addition, many sibling species including the *An. gambiae *complex are outcomes of recent speciation processes associated with paracentric inversions involving this chromosome [33]. The *X *chromosome banding patterns and inversion frequencies studies of Brazilian south and south-east *An. cruzii *populations, showed three *X *chromosomal forms (A, B and C), suggesting a process of incipient speciation [9,10]. The authors observed that the majority of mosquitoes from Juquitiba population had form A, while form B predominated in Cananéia [9,10]. In the current study, although there are no fixed differences in the *cpr *gene between Juquitiba and Cananéia, a moderately high *F_ST _*value was observed. In *An. gambiae*, *cpr *is located on the *X *chromosome. Therefore, if this molecular marker has a similar chromosomal location in *An. cruzii*, it might be associated with the chromosomal forms described by Ramirez & Dessen [9,10].

Comparisons of the *F_ST _*values observed with *cpr *and *timeless *in all pair-wise comparisons involving the five populations analysed in the current study and in Rona *et al *[14] show only a partial consistence but this is expected. Wang-Sattler *et al *[34] demonstrated that the phylogenetic relationships in the *An. gambiae *complex could vary widely between different genomic regions, thus indicating the mosaic nature of the genome of these species [34].

As mentioned above the *cpr *gene in *An. gambiae *is *X*-linked, while *timeless *is autosomal. Assuming these two markers have similar locations in *An. cruzii*, *cpr *is expected to be under more efficient selection than the *timeless*, since in species with X/Y sex determination, as *An. cruzii*, rare recessive mutations are fully expressed in the heterogametic sex, which could lead to 'faster-*X *evolution' if a large proportion of mutations are fixed by positive selection [35]. If positive selection is more efficient on the *X *chromosome, one expects it to harbour less variability than the autosomes [36]. The *X *chromosome is indeed less variable than the autosomes in non-African populations of *Drosophila simulans *[37]. Comparing *timeless *and *cpr*, the first is more polymorphic than the latter, but the latter shows higher differentiation among the southern populations of *An. cruzii*.

Since *An. cruzii *is polymorphic for chromosomal inversions and Ramirez & Dessem [9,10] found evidence for sibling species carrying different *X *chromosomal forms, another hypothesis that might explain the differences between the two markers is the suppressed-recombination model of speciation proposed by Coluzzi [33,38].

Analysis of the molecular polymorphism and genetic differentiation of the *timeless *gene among Brazilian populations of *An. cruzii *suggested that the population from Itatiaia (Rio de Janeiro State) is in a process of differentiation and incipient speciation [14]. High *F_ST _*values between Itatiaia (Rio de Janeiro State) and the other populations from south and south-east Brazil was reported here. In addition, comparison of Itatiaia with other populations revealed some fixed differences and only a few shared polymorphisms. Moreover, the haplotype network shows that Itatiaia is clearly separated in an isolated group (Figure 3). These results, therefore, suggest that this population represents a different species in the *An. cruzii *complex.

Preliminary evidence was also presented here that raised the possibility of the existence of two different sympatric incipient species in Itatiaia. This is based on the analysis of the genetic differentiation of the *cpr *gene and a reanalysis of the recently published *timeless *data [14]. Although a putative heterozygote was found in *cpr *analysis considering the AG repeats and shared polymorphisms were observed in *timeless*, high *F_ST _*values were detected between Itatiaia A and Itatiaia B in these two molecular markers, as well as fixed differences which seem to indicate that these two groups might represent different incipient species. Inspection of the neighbour-joining tree presented in the *timeless *study [14] reveals that the individuals classified here as Itatiaia A are clearly isolated in a separated branch. However, the individuals classified as Itatiaia B are mixed with the other individuals from south and south-east populations. In that study, only one putative heterozygote was found (Ita01) carrying alleles of the two Itatiaia groups. Unfortunately this DNA sample was lost and therefore it was not possible to analyse the *cpr *gene of this individual mosquito. The sample sizes available for the two Itatiaia groups are quite small and further work is clearly needed to determined beyond any doubt that two sympatric incipient sibling species exist in this locality but the results presented here seems to indicate that might be the case.

Analysis of a number of other molecular markers will allow a more precise estimate of the Itatiaia population differentiation and might provide a more complete representation of the divergence history of this species complex.

## Conclusions

Evidence was presented here suggesting the existence of at least one new sibling species within the *Anopheles *(*Kerteszia*) *cruzii *complex in Itatiaia, south-east Brazil, a finding that supports a previous *timeless *gene study. In addition, according to *cpr *and *timeless *gene analyses, the Itatiaia sample might be in fact composed by two sympatric incipient species, named here Itatiaia A and Itatiaia B.

## Competing interests

The authors declare that they have no competing interests.

## Authors' contributions

LDPR participated in data generation and analysis, and drafted the manuscript. She also helped capture mosquitoes in Florianópolis. CJCP carried out the capture and morphological identification of mosquitoes collected in Florianópolis. AAP is the principal investigator, participated in its design and coordination, and helped to write the manuscript. All authors read and approved the final manuscript.

## Supplementary Material

Additional file 1 **CPR protein multiple alignment and primer positions**. The putative fragment of *An. cruzii *CPR deduced protein is aligned with *D. melanogaster*, *D. pseudoobscura*, *M. domestica*, *An. gambiae *and *Ae. aegypti *homologues. Arrows point to the approximated positions of the primers used in this study. The inverted triangle represents the position of the intron. Click here for file

Additional file 2 **Alignment of the *An. cruzii cpr *sequences**. Alignment of the DNA sequences from the *cpr *gene fragment from all populations of *An. cruzii *analysed. The translated amino acid sequence is shown above the alignment and the intron is highlighted in grey. Dots represent identity and dashed represent gaps. The asterisks in the bottom line represent identity of all sequences. Flo: individuals from Florianópolis; Can: Cananéia; Juq: Juquitiba; Ita: Itatiaia; San: Santa Teresa. Click here for file

Additional file 3 **Haplotype network using *timeless *nucleotide sequences of the Itatiaia population**. Each circle represents a different haplotype with size proportional to its relative frequency. Haplotype numbers are given in Roman and the number of sequences of each haplotype is given in brackets. The small white circles represent missing intermediates and the lines connecting the haplotypes represent one mutational step between two observed haplotypes. Each individual of Itatiaia population is discriminated next to respective haplotype. Click here for file
