# Supplementary material for: Molecular evidence for the occurrence of a new sibling species within the Anopheles (Kerteszia) cruzii complex in south-east Brazil
Source: Malar J. 2010 Jan 26;9:33. doi: 10.1186/1475-2875-9-33 (PMC2825240; doi:10.1186/1475-2875-9-33)
Supplement: Additional file 1 — CPR protein multiple alignment and primer positions. The putative fragment of An. cruzii CPR deduced protein is aligned with D. melanogaster, D. pseudoobscura, M. domestica, An. gambiae and Ae. aegypti homologues. Arrows point to the approximated positions of the primers used in this study. The inverted triangle represents the position of the intron. [file 1475-2875-9-33-S1.DOC]

**3’cpr01ancruzii**

**5’cpr01ancruzii**

**5’Cpr01deg**

**3’Cpr01deg**

*D. melanogaster* LKGMVADPEECDMEELLQLKDIDNSLAVFCLATYGEGDPTDNAM

*D. pseudoobscura* LKGMVADPEECDMEELLQLKDISNSLAVFCLATYGEGDPTDNAM

*M. domestica* MKGMVADPEECDMEELLQMKDIPNSLAVFCLATYGEGDPTDNAM

*Ae. aegypt* MKDMVADPEECDMEELLSLKDIDKSLAVFCLATYGEGDPTDNCM

*An. gambiae* MKGMVADPEECNMEELLMLKDIDKSLAVFCLATYGEGDPTDNCM

*An. cruzii* MKGMVADPEECNMEELLQLKDIEKSLAVFCLATYGEGDPTDNCM

:*.********:***** :*** :******************.*
