# Supplementary material for: Molecular evidence for the occurrence of a new sibling species within the Anopheles (Kerteszia) cruzii complex in south-east Brazil
Source: Malar J. 2010 Jan 26;9:33. doi: 10.1186/1475-2875-9-33 (PMC2825240; doi:10.1186/1475-2875-9-33)
Supplement: Additional file 2 — Alignment of the An. cruzii cpr sequences. Alignment of the DNA sequences from the cpr gene fragment from all populations of An. cruzii analysed. The translated amino acid sequence is shown above the alignment and the intron is highlighted in grey. Dots represent identity and dashed represent gaps. The asterisks in the bottom line represent identity of all sequences. Flo: individuals from Florianópolis; Can: Cananéia; Juq: Juquitiba; Ita: Itatiaia; San: Santa Teresa. [file 1475-2875-9-33-S2.DOC]

### 0000000000000000000000000000000000000000000000000000000000000000000000000000000000000000000000000001111111111111111111111111111111111111111111111111111111111111111111111111111111111111

### 0000000001111111111222222222233333333334444444444555555555556666666667777777777888888888899999999990000000000111111111122222222223333333333444444444455555555556666666666777777777788888

1234567890123456789012345678901234567890123456789012345678901234567890123456789012345678901234567890123456789012345678901234567890123456789012345678901234567890123456789012345678901234

C N M E E L L Q L K D I E K

### Flo04a AGTGTAATATGGTAAGCGAA-CGCG--AGAGAGAGAG------------AGTCCGCGCCTATACGGTGACGCCGGCGGGCGGGCGGG----CCAGCATGTTGTAATCCGTTCCGTTCCACTCTCTCTCTCTCTCTCTGTGCACACGCAGGAAGAGCTGTTGCAGCTGAAAGACATCGAGAAATC

### Flo04b ....................-....--..........------------......................................----............................................--...............................................

### Flo05a ....................-....--..........AGAGAGAGAG--.................................C-...CGGG..........................................----...............................................

### Flo05b ....................-....--..........------------......................................----............................................--...............................................

### Flo06a ....................-....--..........------------......................................----............................................--...............................................

### Flo06b ....................-....--..........------------......................................----............................................--...............................................

### Flo07a ....................-....--..........------------......................................----............................................--...............................................

### Flo07b ....................-....--..........------------......................................----............................................--...............................................

### Flo08a ....................-....--..........------------......................................----............................................--...............................................

### Flo08b ....................-....--..........------------......................................----..........................................----...............................................

### Flo09a ....................-....--..........------------......................................----............................................--...............................................

### Flo09b ....................-....--..........------------......................................----............................................--...............................................

Flo10a ....................-....--..........------------......................................----............................................--...............................................

Flo10b ....................-....--..........------------......................................----............................................--...............................................

Flo11a ....................-....--..........AGAGAGAG----.A...............................C-...CGGG..........................................----...............................................

Flo11b ....................-....--..........------------......................................----............................................--...............................................

Flo12a ....................-....--..........------------......................................----.............................................................................................

Flo12b ....................-....--..........------------......................................----............................................--...............................................

Flo13a ....................-....--..........------------......................................----............................................--...............................................

Flo13b ....................-....--..........------------......................................----............................................--...............................................

Flo15a ....................-....--..........------------......................................----............................................--...............................................

Flo15b ....................-....--..........------------......................................----............................................--...............................................

Flo16a ....................-....--..........------------......................................----............................................--...............................................

Flo16b ....................-....--..........------------......................................----............................................--...............................................

Flo17a ....................-....--..........------------......................................----............................................--...............................................

Flo17b ....................-....--..........------------......................................----............................................--...............................................

Flo18a ....................-....--..........AGAGAGAGAG--.A...............................C-...CGGG........................................------...............................................

Flo18b ....................-....--..........AGAGAGAGAGAG.A...............................C-...CGGG..........................................----...............................................

Can01a ....................-....--..........------------......................................----.............................................................................................

Can01b ....................-....--..........------------......................................----............................................--...............................................

Can02a ....................-....--..........AGAGAGAG----.................................C-...CGGG..........................................----...............................................

Can02b ....................-....--..........AGAGAGAGAGAG.................................C-...CGGG..........................................----...............................................

Can03a ....................-....--..........------------......................................----..........................................----...............................................

Can03b ....................-....--..........------------......................................----..........................................----...............................................

Can04a ....................-....--..........------------......................................----............................................--...............................................

Can04b ....................-....--..........------------.................................C-...CGGG..........................................----...............................................

Can05a ....................-....--..........------------......................................----............................................--...............................................

Can05b ....................-....--..........------------......................................----............................................--...............................................

Can06a ....................-....--..........AGAGAGAG----.................................C-...CGGG..........................................----...............................................

Can06b ....................-....--..........AGAGAGAG----.................................C-...CGGG..........................................----...............................................

Can07a ....................-....--..........------------......................................----..........................................----...............................................

Can07b ....................-....--..........------------......................................----............................................--...............................................

Can08a ....................-....--..........------------......................................----..........................................----...............................................

Can08b ....................-....--..........------------......................................----............................................--...............................................

Can09a ....................-....--..........------------......................................----............................................--...............................................

Can09b ....................-....--..........------------......................................----............................................--...............................................

Can10a ....................-....--..........AGAGAGAG----......................................----............................................--...............................................

Can10b ....................-....--..........AGAGAGAGAGAG...............................A.C-...CGGG..........................................----...............................................

Can11a ....................-....--..........------------......................................----............................................--...............................................

Can11b ....................-....--..........AG----------.................................C-...CGGG..........................................----...............................................

Can12a ....................-....--..........AGAGAGAGAGAG.................................C-...CGGG..........................................----...............................................

Can12b ....................-....--..........AGAGAGAG----.................................C-...CGGG..........................................----...............................................

Juq02a ....................-....--...A......------------.................................C....----..........................................----...............................................

Juq02b ....................-....--...A......------------.................................C....----..........................................----...............................................

Juq11a ....................-....--..........AG----------.................................C....----..........................................----...............................................

Juq11b ....................-....--..........------------.................................C....----..........................................----...............................................

Juq19a ....................-....--..........------------.................................C....----..........................................----...............................................

Juq19b ....................-....--..........------------.................................C....----..........................................----...............................................

Juq34a ....................-....--..........------------.................................C....----..........................................----...............................................

Juq34b ....................-....--..........------------.................................C....----..........................................----...............................................

Juq35a ....................-....--..........AGAGAGAG----....................T............C....----..........................................----...............................................

Juq35b ....................-....--..........------------.................................C....----..........................................----...............................................

Juq42a ....................-....--..........------------.................................C....----..........................................----...............................................

Juq42b ....................-....--..........------------.................................C....----..........................................----...............................................

Juq43a ....................-....--..........------------.................................C....----..........................................----...............................................

Juq43b ....................-....--..........------------......................................----..........................................----...............................................

Juq50a ....................-....--..........------------.................................C....----..........................................----...............................................

Juq50b ....................-....--..........------------.................................C....----..........................................----...............................................

Juq51a ....................-....--..........------------.................................C....----..........................................----...............................................

Juq51b ....................-....--..........------------.................................C....----..........................................----...............................................

Juq58a ....................-....--..........------------.................................C....----..........................................----...............................................

Juq58b ....................-....--..........------------.................................C....----..........................................----...............................................

Juq67a ....................-....--..........------------.................................C....----............................................--...............................................

Juq67b ....................-....--..........------------.................................C....----..........................................----...............................................

Juq69a ....................-....--..........------------.................................C....----..........................................----...............................................

Juq69b ....................-....--..........AG----------.................................C....----..........................................----...............................................

San01a ....................-....--..........------------.................................C....----....T.....................................----...............................................

San01b ....................-....CG........--------------.....................A................----............................................--...............................................

San02a ....................-....--........--------------.................................C....----............................................--...............................................

San02b ....................-....--..........------------.................................C....----..........................................----...............................................

San03a ....................-....--..........------------.................................C....----..........................................----...............................................

San03b ....................-....CG........--------------.....................A........T.......----............................................--...............................................

San04a ....................-....--..........AGAGAG------......................................----..........................................----........A......................................

San04b ....................-....--..........AGAGAGAGAGAG......................................----..........................................----........A......................................

San06a ....................-....--..........------------.................................C....----..........................................----...............................................

San06b ....................-....--..........------------.................................C....----..........................................----...............................................

San07a ....................-....CG........--------------.....................A........T.......----............................................--...............................................

San07b ....................-....CG........--------------.....................A........T.......----............................................--...............................................

San14a ....................-....CG........--------------.....................A........T.......----............................................--...............................................

San14b ....................-....CG........--------------.....................A................----............................................--...............................................

Ita02a ....................A..----..........AGAG--------................T.................--------....................................----------...............................................

Ita02b ....................A..----..........AGAGAG------..................................--------....................................----------...............................................

Ita03a ....................A..----..........AGAGAG------..................................--------....................................----------...............................................

Ita03b ....................A..----..........AGAG--------..................................--------....................................----------...............................................

Ita04a ....................A..----..........AG----------..................................--------....................................----------...............................................

Ita04b ....................A..----..........AGAG--------..................................--------....................................----------...............................................

Ita05a ....................A..----..........------------......................................----....................................----------...............................................

Ita05b ....................A..----..........------------......................................----....................................----------...............................................

Ita06a ....................A..----..........------------......................................----....................................----------...............................................

Ita06b ....................A..----..........------------......................................----....................................----------...............................................

Ita07a ....................A..----..........------------........T..........T................A.----......................A.............----------...............................................

Ita07b ....................A..----..........------------........T..........T................A.----......................A.............----------...............................................

Ita08a ....................A..----..........AGAG--------................T.................--------....................................----------...............................................

Ita08b ....................A..----..........AGAGAG------..................................--------....................................----------...............................................

Ita09a ....................A..----..........------------.....................................C----......................A.............----------...............................................

Ita09b ....................A..----..........------------......................................----....................................----------...............................................

Ita10a ....................A..----..........AGAG--------................T.................--------....................................----------...............................................

Ita10b ....................A..----..........AGAG--------..................................--------....................................----------...............................................

Ita11a ....................A..----..........AGAG--------................T.................--------....................................----------...............................................

Ita11b ....................A..----..........AGAGAG------..................................--------....................................----------...............................................

Ita12a ....................A..----..........------------.....................................C----......................A.............----------...............................................

Ita12b ....................A..----..........AGAGAG------..................................--------....................................----------...............................................

******************** ** *** **** * ****** ******* ** ******** * **** ***************** ************* ******** **************************************
