# Supplementary material for: Molecular evidence for the occurrence of a new sibling species within the Anopheles (Kerteszia) cruzii complex in south-east Brazil
Source: Malar J. 2010 Jan 26;9:33. doi: 10.1186/1475-2875-9-33 (PMC2825240; doi:10.1186/1475-2875-9-33)
Supplement: Additional file 3 — Haplotype network using timeless nucleotide sequences of the Itatiaia population. Each circle represents a different haplotype with size proportional to its relative frequency. Haplotype numbers are given in Roman and the number of sequences of each haplotype is given in brackets. The small white circles represent missing intermediates and the lines connecting the haplotypes represent one mutational step between two observed haplotypes. Each individual of Itatiaia population is discriminated next to respective haplotype. [file 1475-2875-9-33-S3.PPT]

## Slide 1
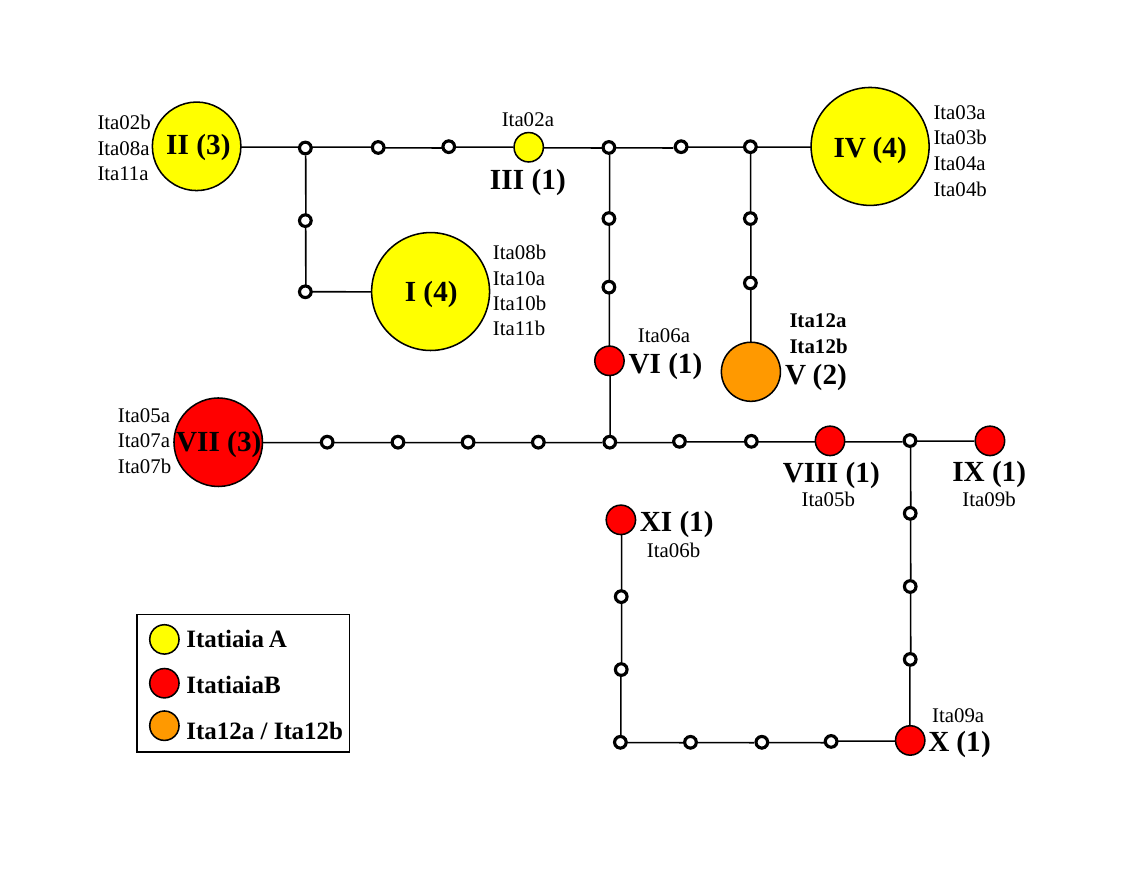

Ita03a
Ita03b
Ita04a
Ita04b
Ita02a
Ita02b
Ita08a
Ita11a
II (3)
IV (4)
III (1)
Ita08b
Ita10a
Ita10b
Ita11b
I (4)
Ita12a
Ita12b
Ita06a
VI (1)
V (2)
Ita05a
Ita07a
Ita07b
VII (3)
IX (1)
VIII (1)
Ita05b
Ita09b
XI (1)
Ita06b
Ita09a
X (1)
Itatiaia A
ItatiaiaB
Ita12a / Ita12b
